# High-fat diet feeding differentially affects the development of inflammation in the central nervous system

**DOI:** 10.1186/s12974-016-0666-8

**Published:** 2016-08-26

**Authors:** Owein Guillemot-Legris, Julien Masquelier, Amandine Everard, Patrice D. Cani, Mireille Alhouayek, Giulio G. Muccioli

**Affiliations:** 1 Bioanalysis and Pharmacology of Bioactive Lipids Research Group, Louvain Drug Research Institute (LDRI), Université catholique de Louvain (UCL), Av. E.Mounier, 72 (B1.72.01), 1200 Brussels, Belgium; 2 Metabolism and Nutrition Research Group, WELBIO - Walloon Excellence in Life Sciences and BIOtechnology, Louvain Drug Research Institute, Université catholique de Louvain, Brussels, Belgium

**Keywords:** Obesity, Bioactive lipid, Astrocyte, Microglia, HPLC-MS, Cortex, Cerebellum, PEA

## Abstract

**Background:**

Obesity and its associated disorders are becoming a major health issue in many countries. The resulting low-grade inflammation not only affects the periphery but also the central nervous system. We set out to study, in a time-dependent manner, the effects of a high-fat diet on different regions of the central nervous system with regard to the inflammatory tone.

**Methods:**

We used a diet-induced obesity model and compared at several time-points (1, 2, 4, 6, 8, and 16 weeks) a group of mice fed a high-fat diet with its respective control group fed a standard diet. We also performed a large-scale analysis of lipids in the central nervous system using HPLC-MS, and we then tested the lipids of interest on a primary co-culture of astrocytes and microglial cells.

**Results:**

We measured an increase in the inflammatory tone in the cerebellum at the different time-points. However, at week 16, we evidenced that the inflammatory tone displayed significant differences in two different regions of the central nervous system, specifically an increase in the cerebellum and no modification in the cortex for high-fat diet mice when compared with chow-fed mice. Our results clearly suggest region-dependent as well as time-dependent adaptations of the central nervous system to the high-fat diet. The differences in inflammatory tone between the two regions considered seem to involve astrocytes but not microglial cells. Furthermore, a large-scale lipid screening coupled to ex vivo testing enabled us to identify three classes of lipids—phosphatidylinositols, phosphatidylethanolamines, and lysophosphatidylcholines—as well as palmitoylethanolamide, as potentially responsible for the difference in inflammatory tone.

**Conclusions:**

This study demonstrates that the inflammatory tone induced by a high-fat diet does not similarly affect distinct regions of the central nervous system. Moreover, the lipids identified and tested ex vivo showed interesting anti-inflammatory properties and could be further studied to better characterize their activity and their role in controlling inflammation in the central nervous system.

**Electronic supplementary material:**

The online version of this article (doi:10.1186/s12974-016-0666-8) contains supplementary material, which is available to authorized users.

## Background

Obesity and related disorders are becoming worldwide health issues [1–3]. Obesity is regarded as an inflammatory condition because of the associated low-grade inflammation [4–6] affecting the periphery and increasing the incidence of many pathologies such as cardiovascular diseases [7], asthma [8], or even cancer [9]. One of the proposed mechanisms leading to peripheral inflammation implicates the gut microbiota. More specifically, a high-fat diet (HFD) will change the balance between different populations of bacteria within the gut [10, 11]. This will lead to a disruption of the intestinal epithelium integrity that in turn will result in increased passage of endotoxins (such as lipopolysaccharides (LPS)) into the bloodstream that will then fuel the peripheral inflammatory tone [4, 12, 13]. The demonstration that disrupting LPS signaling (i.e., TLR4^−/−^ mice or CD14^−/−^ mice) protects from diet-induced obesity and metabolic disorders strongly supports the important role played by LPS in the pathophysiology of these disorders [12–15].

This increased peripheral inflammatory tone will also affect the central nervous system (CNS) and will increase the incidence of CNS pathologies such as cognitive impairments [16], Alzheimer’s disease [17], stroke [18], or dementia [19]. The impact of a HFD on the CNS was well characterized with regard to a specific region, the hypothalamus [20–22]. The hypothalamus has attracted the attention of many researchers because of its central role in food intake as well as in monitoring the availability of nutrients [23–25]. HFD feeding is associated with a disruption of the homeostasis in the hypothalamus, and more specifically with the activation of glial cells and increased inflammatory tone [24, 26, 27]. This leads to both leptin and insulin resistance thus worsening obesity [22]. However, much less is known about the repercussions of a HFD on the other regions of the CNS in terms of inflammation. Obesity and inflammation are closely related to lipids and their metabolism. Indeed, HFD feeding will lead to an increase in the intake of saturated fatty acids [28] and to the disruption of cholesterol homeostasis (an increase in LDL to HDL cholesterol ratio) [29, 30], both associated with deleterious effects. The adipose tissue will have to cope with an increased flow of free fatty acids that will trigger a low-grade inflammation through immunomodulatory changes of both specific T cell subtypes and macrophage polarization [31, 32].

The perception of lipids has dramatically changed from being mere energy substrate molecules to bioactive molecules involved in many physiological processes notably through the emergence of the lipidomic approach [33]. Lipids are recognized as central mediators involved in the onset, development, and resolution of inflammatory processes [34, 35]. Obesity alters the endogenous levels of several bioactive lipid families such as ceramides, phosphatidylcholines, and endocannabinoids [36–38]. In turn, some bioactive lipids exert either pro- or anti-inflammatory effects during obesity. For instance, ceramides will exert pro-inflammatory effects in the liver and will progressively lead to insulin resistance by tampering with the insulin signaling [6, 39, 40]. Conversely, n−3 polyunsaturated fatty acids show beneficial effects by counteracting HFD-induced adipose tissue inflammation [41]. Still, the potential involvement of other lipids needs to be addressed to better characterize the inflammatory tone deriving from obesity.

In this study, we set out to characterize, at multiple time-points and in different CNS regions, the inflammatory tone induced by a HFD. We found that, depending on the CNS region, a HFD differentially affects the inflammatory tone. We, therefore, investigated whether changes in CNS lipid content could explain the differences in the inflammatory tone between CNS regions.

## Methods

### Animals and diets

Nine-week-old male C57BL/6J mice (Charles River) were housed in a controlled environment (12-h day light cycle, lights off at 6 pm, controlled temperature and humidity). Upon arrival, they were randomly split into 12 groups of eight mice each (four mice/cage) and acclimated for 1 week. Then, six of these groups were given free access to a standard diet (AIN 93-M, Research Diets, New Brunswick, USA) and the remaining six groups were given free access to a HFD (D12492, Research Diets, New Brunswick, USA). For details in the composition of both diets, refer to Additional file 1: Table S1. For this experiment, we euthanized at each selected time-point (i.e., after 1, 2, 4, 6, 8, and 16 weeks) one group under standard diet and one group under a HFD. Mice were anesthetized using isoflurane after a 6-h fasting period and sacrificed by cervical dislocation. The cortex, cerebellum, and brainstem were carefully and rapidly recovered and snap-frozen in liquid nitrogen. The different adipose tissue depots (subcutaneous adipose tissue (SAT), visceral adipose tissue (VAT), epididymal adipose tissue (EAT), and brown adipose tissue (BAT)) were harvested and weighed. All the tissues collected were stored at −80 °C until further analysis.

We performed this study in accordance with the European recommendation 2007/526/CE (which was transformed into the Belgian Law of May 29, 2013), regarding the protection of laboratory animals. The local ethics committee approved the protocol of the study (study agreement 2010/UCL/MD/022; lab agreement LA1230314).

### Cholesterol quantification

Plasma total cholesterol was quantified, following manufacturer’s instructions, in the vena cava using the Cholesterol FS10 kit (DiaSys Diagnostic and Systems, Holzheim, Germany), which is based on an enzymatic reaction coupled with a spectrophotometric detection of the end-product.

### RNA preparation and RT-qPCR analysis

Total RNA from tissues was extracted using TriPure reagent (Roche, Basel, Switzerland) according to the manufacturer’s instructions. cDNA was synthesized using an RT kit (Promega, GoScript™ Reverse Transcription System) from 1 μg of total RNA. qPCR was performed with a StepOnePlus instrument and software (Applied Biosystems, Foster City, CA, USA). PCR reactions were run using a SYBR Green mix (Promega, GoTaq® qPCR Master Mix). We measured each sample in duplicate during the same run. The following conditions were used for amplification: an initial holding stage of 10 min at 95 °C, then 45 cycles consisting of denaturation at 95 °C for 3 s, annealing at 60 °C for 26 s, and extension at 72 °C for 10 s. Products were analyzed by performing a melting curve at the end of the PCR reaction. Data are normalized to the 60S ribosomal protein L19 (RPL19) messenger RNA (mRNA) expression [42]. The sequences of the primers used are listed in Table 1.

**Table 1:** Primer sequences

Gene	Forward primer (5′–3′)	Reverse primer (5′–3′)
CD11b	GAACATCCCATGACCTTCCA	GCTGGGGGACAGTAGAAACA
CD11c	ACGTCAGTACAAGGAGATGTTGGA	ATCCTATTGCAGAATGCTTCTTTACC
CD68	CTTCCCACAGGCAGCACAG	AATGATGAGAGGCAGCAAGAGG
Claudin 1	TTCGCAAAGCACCGGGCAGATACA	GCCACTAATGTCGCCAGACCTGAAA
Claudin 5	GTTAAGGCACGGGTAGCACT	GTACTTCTGTGACACCGGCA
COX-2	TGACCCCCAAGGCTCAAATAT	TGAACCCAGGTCCTCGCTTA
F4/80	TGACAACCAGACGGCTTGTG	CAGGCGAGGAAAAGATAG
GFAP	TTCGCACTCAATACGAGGCA	CTCCAGATCGCAGGTCAAG
IL-1β	TCGCTCAGGGTCACAAGAAA	CATCAGAGGCAAGGAGGAAAAC
IL-6	ACAAGTCGGAGGCTTAATTACACAT	TTGCCATTGCACAACTCTTTTC
iNOS	AGGTACTCAGCGTGCTCCAC	GCACCGAAGATATCTTCATG
LBP	AGTCCTGGGAATCTGTCCTTG	ACTTGTGCCTTGTCTGGATG
MCP-1	GCAGTTAACGCCCCACTCA	TCCAGCCTACTCATTGGGATCA
Occludin	ATGTCCGGCCGATGCTCTC	TTTGGCTGCTCTTGGGTCTGTAT
RPL19	TGACCTGGATGAGAAGGATGAG	CTGTGATACATATGGCGGTCAATC
Serpina3n	GGACATTGATGGTGCTGGTGAAT	CTCCTCTTGCCCGCGTAGAA
TNFα	CCACCACGCTCTTCTGTCT	TCCAGCTGCTCCTCCACTT
ZO-1	TTTTTGACAGGGGGAGTGG	TGCTGCAGAGGTCAAAGTTCAAG

### Lipid quantification

Tissues (cerebellum or cortex) were homogenized in water (2.5 mL), and then the lipids were extracted following acidification, in the presence of internal standards, by adding 10 mL of chloroform (CHCl_3_) and 5 mL of methanol (MeOH). Following vigorous mixing and sonication, the samples were centrifuged and the organic layer was recovered and dried under a stream of N_2_. The resulting lipid extracts were purified by solid-phase extraction using silica and eluted with a mix of CHCl_3_ and MeOH. The resulting lipid fractions were analyzed by HPLC-MS using an LTQ-Orbitrap mass spectrometer (ThermoFisher Scientific) coupled to an Accela HPLC system (ThermoFisher Scientific). Analyte separation was achieved using a C-18 Phenomenex pre-column and a Kinetex LC-18 column (5 μm, 4.6 × 150 mm) (Phenomenex).

For lysophosphosphatidylcholines, phosphatidylcholines, and sphingomyelins, mobile phases A and B were composed of MeOH-H_2_O 85:15 (*v*/*v*) and MeOH, respectively, with 5 mM of CH_3_COONH_4_. The gradient (0.25 mL/min) was designed as follows: transition from 100 % A to 100 % B over 15 min, followed by 100 % B linearly over 15 min followed by a subsequent re-equilibration at 100 % A. Analytes were ionized using an ESI source operated in positive mode.

For the other lysophosphospholipids and for the phospholipids and sulfatides, mobile phases A and B were composed of MeOH-H_2_O-NH_4_OH 50:50:0.1 (*v*/*v*/*v*) and MeOH-NH_4_OH 100:0.1 (*v*/*v*), respectively. The gradient (0.4 mL/min) was designed as follows: transition from 100 % A to 100 % B over 30 min, followed by 100 % B linearly over 15 min, and followed by a subsequent re-equilibration at 100 % A. Analytes were ionized using an ESI source operated in negative mode.

For *N*-acylethanolamines and ceramides, mobile phases A and B were composed of MeOH-H_2_O-acetic acid 75:25:0.1 (*v*/*v*/*v*) and MeOH-acetic acid 100:0.1 (*v*/*v*), respectively. The gradient (0.4 mL/min) was designed as follows: transition from 100 % A to 100 % B over 15 min, followed by 100 % B linearly over 45 min, followed by a subsequent re-equilibration at 100 % A. Analytes were ionized using an APCI source operated in positive mode. The signals of the lipids were normalized using the signal obtained for the corresponding internal standard. We used d_4_-PEA, 17:1-lysophosphatidylinositol, 17:0-lysophosphatidylcholine, 17:0-sulfatide, 17:0-ceramide, 17:0-sphingomyelin, and 17:0/17:0-PC. Data are presented as fold increase compared with levels found in control mice.

### Immunohistology

During the sacrifice, sections of the cortex and cerebellum were transferred to a solution of 4 % PFA in PBS and stored at 4 °C for 24 h. Cryopreservation was performed by incubation in a solution of 20 % sucrose in PBS for a further 24 h at 4 °C. Finally, tissues were embedded in Tissue-Tek (Sakura Finetek, Zoeterwoude, The Netherlands) and kept at −80 °C. Sections were cut (30 μm) using a cryostat and then used for the detection of microglial cells (Iba-1) and astrocytes (GFAP). The sections were incubated in blocking solution containing 5 % normal donkey serum and 1 % Triton X-100 (Sigma-Aldrich, Seelze, Germany) in PBS for 60 min. The primary antibodies, rabbit anti-Iba1 (Wako Laboratory Chemicals, Japan) (1:1000 in PBS/triton 1 %) and direct rat anti-GFAP (1:250 in PBS/triton 1 %), were applied for 12 h at 4 °C. Tissues were then rinsed three times with PBS. The secondary antibody anti-rabbit Alexa 488 (Thermo Fisher Scientific) (1:100) was applied for 1 h at room temperature. Tissues were washed with PBS and nuclei were stained using Hoescht. Slides were mounted using Dako Fluorescence Mounting Medium. Stained slides were digitized using a Mirax Midi scanner (Carl Zeiss Micro-Imaging). Image acquisition was executed with Mirax Scan software (Zeiss). The obtained images were analyzed (by a researcher blinded to the treatment) using ImageJ software (http://imagej.nih.gov/ij/) and/or CellProfiler software (http://www.cellprofiler.org/).

### Primary glial cell culture and treatment

C57BL/6J mice pups (post-natal day 2–3) were euthanized, the brain recovered, and their cerebral cortices dissected. Tissues were then mechanically dissociated by several sequences of pipetting and sedimenting, then centrifuged and resuspended in DMEM-F12 media (containing 10 % FBS and 100 units/mL of penicillin and 100 μg/mL of streptomycin). Cells were seeded in poly-lysine pre-coated flasks (two pups per flask) and incubated for 2 weeks with two media changes at day 5 and 10. After 14 days of culture, cells were trypsinized and secondary cultures were seeded overnight in poly-lysine pre-coated 24-well plates (150,000 cells/well). Cells were then incubated with fresh culture medium containing the compounds of interest (10 μM), and LPS (10 ng/mL, from *E. coli* 055:B5) was added 1 h later. After 8 h, the media was removed and Tripure^©^ was added to the cells for mRNA analysis (see above). For all experiments, a control condition was performed where cells were only incubated with vehicle (DMSO, 0.2 %) in the absence of LPS.

### Inflammatory plasma cytokine quantification

Plasma cytokines IL-1β, IL-10, and tumor necrosis factor α (TNFα) were quantified using a Bio-Plex Multiplex kit (Bio-Rad, Nazareth, Belgium) and measured by using Luminex technology (Bio-Plex 200; Bio-Rad) following the manufacturer’s instructions.

### Statistical analysis

All data are presented as mean ± s.e.m. Statistical analysis was performed using GraphPad Prism version 5.0 for Windows (San Diego, CA). We used two-tailed Student’s *t* test for unpaired values to compare two groups, and when relevant, we used the Mann-Whitney test for the peripheral inflammation assessment. We used one-way ANOVA with Bonferroni’s post test or Kruskal-Wallis test with Dunn’s post test between HFD group and its respective CTL group (*P < 0.05; **P < 0.01; and ***P < 0.001) and between CNS regions (#*P* < 0.05; ##*P* < 0.01; and ###*P* < 0.001) for the comparison of inflammatory markers, immunohistological analysis, and the lipid levels for the two CNS areas studied. Finally, we used the one-way ANOVA test with Dunnett’s post test for the ex vivo experiments. For all statistical tests, statistical significance was taken when *P* < 0.05.

## Results and discussion

### Characterization of the obese phenotype

We monitored the weight of mice for the different groups throughout the study. Body weight already increased after 4 days of HFD feeding and persistently increased over time. Conversely, the mice of the control groups did not significantly gain weight (Additional file 2: Figure SI 1). We also weighed the different white adipose tissue depots at each selected time-point. There is a sustained increase of the subcutaneous (SAT), epididymal (EAT), and visceral (VAT) adipose tissues over time (Additional file 2: Figure SI 2). We also measured the cholesterolemia of mice at the earliest (1 week) and latest (16 weeks) time-points. We found a clear increase for mice fed a HFD compared with their respective controls (Additional file 2: Figure SI 3a-b). Taken together, these data validate the obesogenic properties of the diet used.

### Peripheral inflammation induced by HFD feeding

**Fig. 1:**
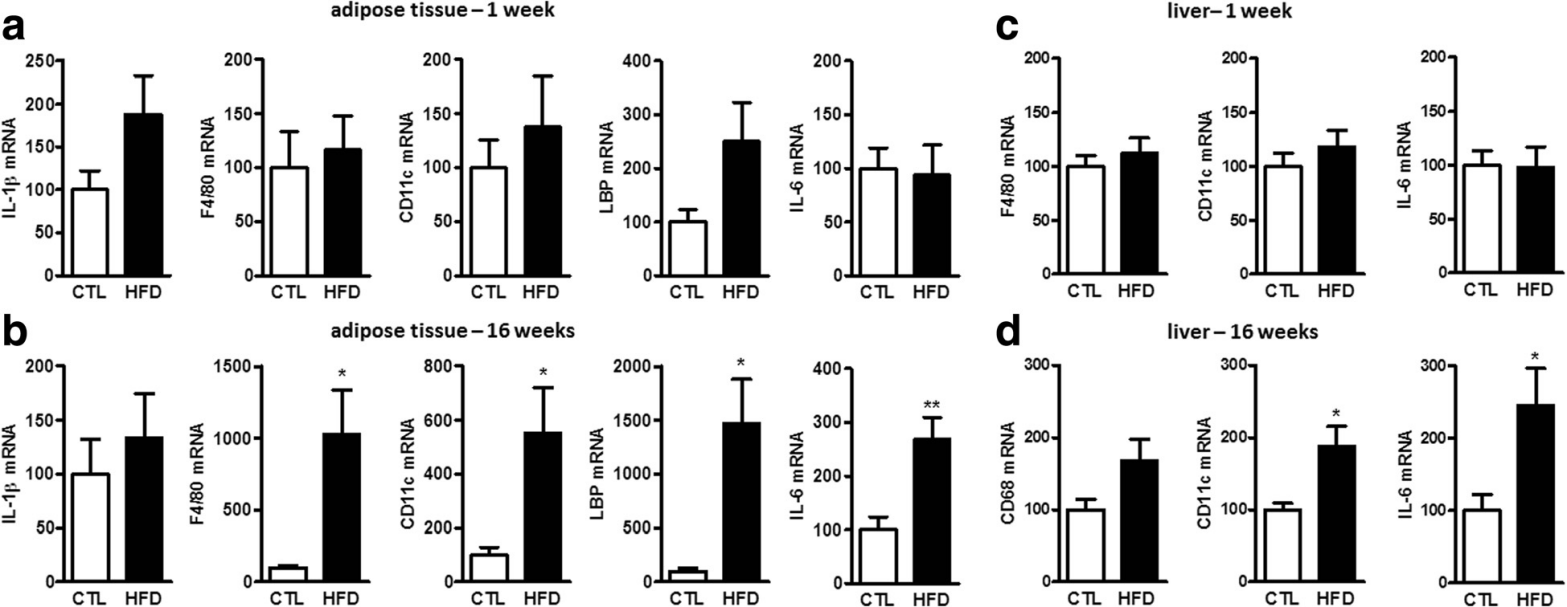
Peripheral inflammatory tone induced by a HFD at weeks 1 and 16. mRNA relative expression of inflammatory and macrophage markers at **a** 1 week and **b** 16 weeks in the subcutaneous adipose tissue (SAT) and mRNA relative expression of inflammatory and macrophage markers at **c** 1 week and **d** 16 weeks in the liver. Results are expressed relative to the control diet group (CTL) set at 100 %. Data are mean ± s.e.m; Student’s *t* test or Mann-Whitney test between HFD group and its CTL group (*P < 0.05 and **P < 0.01)

Because obesity is accompanied by a low-grade peripheral inflammation, we next sought to study the impact of the HFD on the peripheral inflammatory tone. Thus, we measured the mRNA expression of different inflammatory markers in the SAT at weeks 1 and 16 (Fig. 1a, b).. In this tissue, as might be expected for an obesogenic diet, after 16 weeks of HFD, we measured a significant increase in the expression of F4/80 (a macrophage marker), CD11c (a M1 polarization marker), LPS-binding protein (LBP), and interleukin-6 (IL-6) (Fig. 1b). These observations confirm the establishment and progression of an inflammatory tone induced by the HFD in the SAT. Because the liver is another organ that can be affected by inflammation in obese conditions, we also measured the expression of CD68, CD11c, and IL-6 in the liver of mice after 1 and 16 weeks of HFD (Fig. 1c, d). We found a significant increase in the expression of CD11c and IL-6 at 16 weeks (Fig. 1c, d). Finally, we measured the concentration of two pro-inflammatory cytokines (IL-1β and TNFα) and one anti-inflammatory cytokine (IL-10) in the plasma of mice at week 16 and found a significant increase of TNFα for mice fed a HFD compared with chow-fed mice (Additional file 2: Figure SI 3c). Taken together, these data validate the pro-inflammatory effects, in the periphery, of the obesogenic diet used.

### Central inflammation induced by HFD feeding

Obesity is a well-established contributing factor increasing the incidence of peripheral pathologies. It is also well demonstrated that obesity induces inflammation in the hypothalamus [21, 22, 27]. However, much less is known about the effects of obesity on other CNS areas. We thought that two regions, the cerebellum and the cortex, were of particular interest because obesity induces morphological changes in these two areas. Indeed, obese patients display differences in gray matter density in these two specific regions when compared with lean subjects [43, 44]. Early onset obesity is also associated with several cerebellar abnormalities such as neuronal injuries, smaller volume, and compromised development [45, 46]. As for the cortex, it is an area responsible for the cognitive control of food intake [44, 47, 48]. Those specific changes could be either a cause or a consequence of obesity and further maintain dysregulations in food-oriented behaviors.

**Fig. 2:**
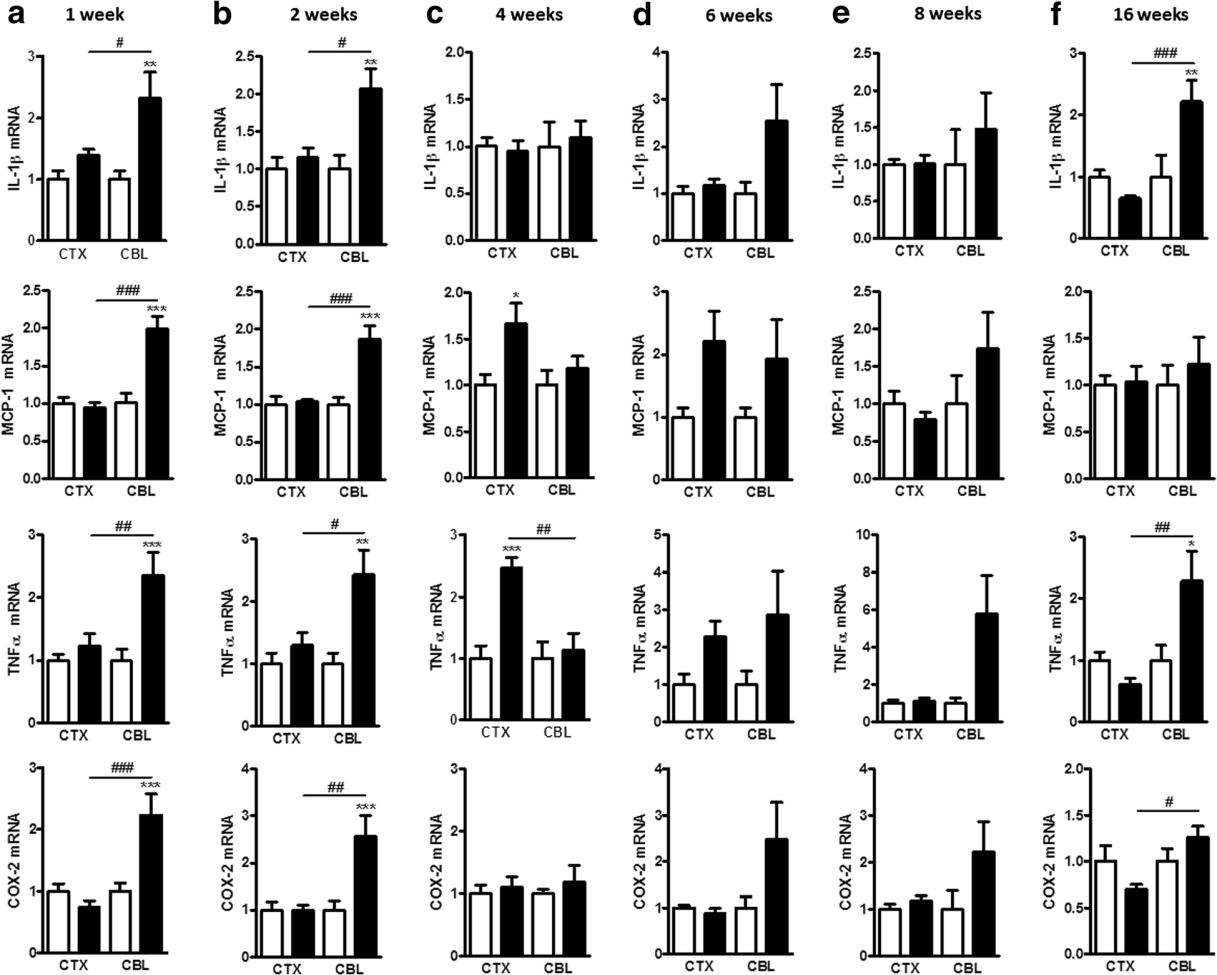
Central inflammatory tone induced by a HFD at different time-points. mRNA expression of inflammatory markers at the different time-points in the cerebellum (CBL) and in the cortex (CTX) at **a** 1 week, **b** 2 weeks, **c** 4 weeks, **d** 6 weeks, **e** 8 weeks, and **f** 16 weeks. Data are mean ± s.e.m. The standard diet groups were set at 1. The *white columns* represent mice fed a standard diet and the *black columns* represent the mice fed a HFD. One-way ANOVA with Bonferroni’s post test or Kruskal-Wallis test with Dunn’s post test between HFD group and its respective CTL group (*P < 0.05; **P < 0.01; and ***P < 0.001) and between CNS regions (#*P* < 0.05; ##*P* < 0.01; and ###*P* < 0.001)

In obesity settings, one major inflammatory pathway affected is the one involving the nuclear factor kappa B (NF-kB). Indeed, this transcription factor is considered as pivotal in the inflammatory tone deriving from obesity, and its involvement in the etiology of metabolic disorders has also been established [49, 50]. Therefore, we set out to study the expression of downstream genes of the NF-kB pathway comprising two cytokines, IL-1β and TNFα as well as the chemokine MCP-1 (known as monocyte chemoattractant protein-1) and the inducible enzyme cyclooxygenase-2 (COX-2). To characterize the changes in inflammatory tone in the cerebellum and cortex during the development of obesity, we measured the mRNA expression of these four inflammatory markers at the six selected time-points of our HFD study (Fig. 2). We found a marked increase of these inflammatory markers in the cerebellum as early as 1 week after HFD feeding. This early inflammation was not present in the cortex. Furthermore, after 16 weeks of HFD feeding, the expression of IL-1β, TNFα, and COX-2 was strikingly different between the two CNS regions considered. Indeed, we found a marked inflammation in the cerebellum of HFD-fed mice when compared with the cortex of the same mice where the inflammatory tone was similar to that of the CTLs.

Because the consequences of the HFD feeding on inflammation were clearly different in the cortex and cerebellum, we sought to determine whether this decreased inflammatory tone was specific to the cortex. Thus, we measured the inflammatory tone in the brainstem, at weeks 1 and 16, and we found an increased expression of IL-1β, TNFα, and MCP-1 at both time-points (Additional file 2: Figure SI 4), a profile similar to the one observed in the cerebellum. Thus, these results clearly suggest region-dependent adaptations of the CNS to the HFD.

To explain these observations, we first assessed the expression of zonula occludens (ZO)-1, claudin 1 and 5, and occludin, four tight junction-forming proteins of the blood-brain barrier [51, 52]. Indeed, the integrity of the blood-brain barrier has been shown to be altered during obesity [53]. We found no variation in the expression of ZO-1 and claudin 5 in the cortex or cerebellum at week 16. However, claudin 1 and occludin expression were significantly increased in the cerebellum, whereas no variation was measured in the cortex at week 16 (Additional file 2: Figure SI 5). Of note, the mRNA expression of these proteins was shown to display consistent variations with proteins detected through immunohistochemistry [54].

**Fig. 3:**
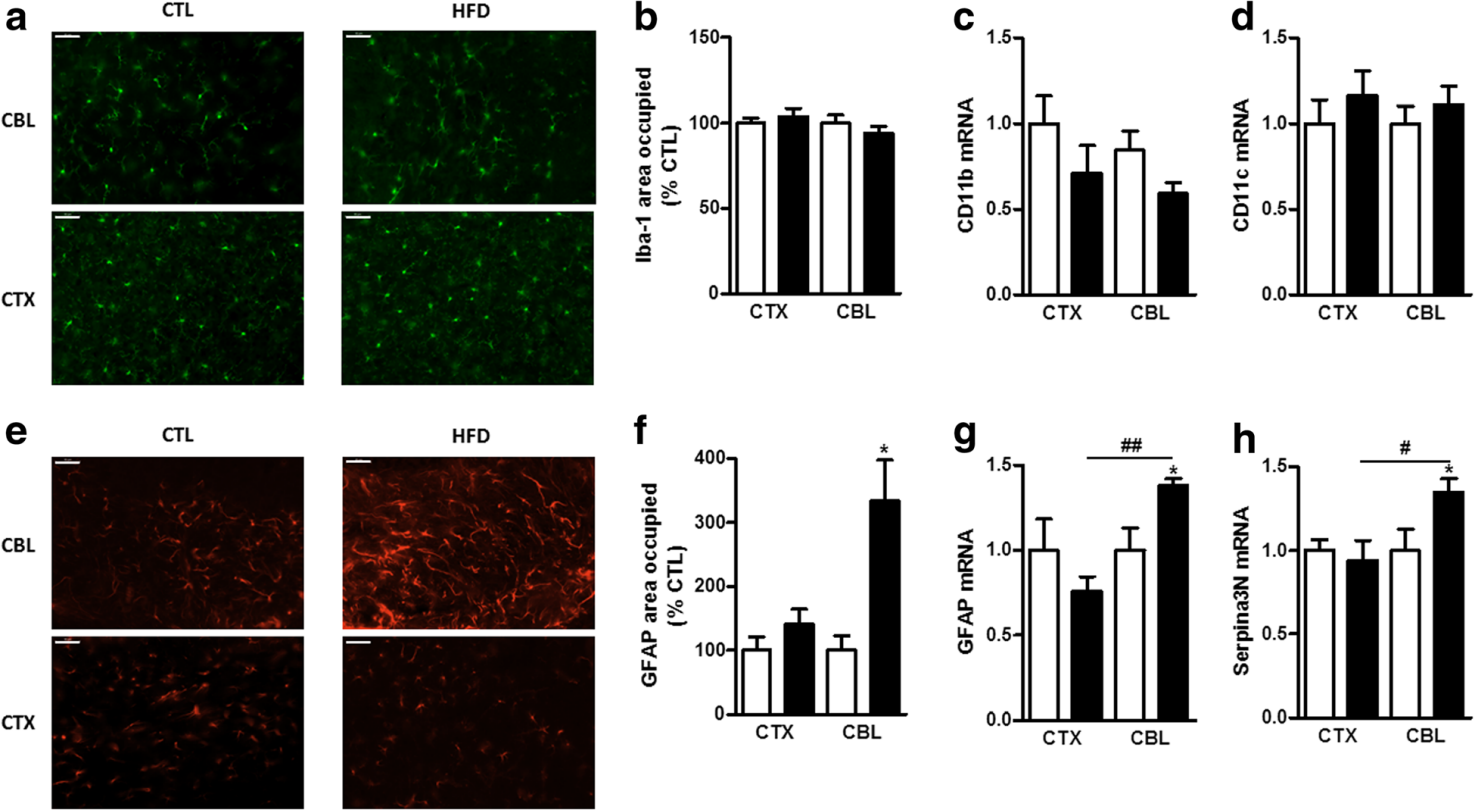
Activation state of glial cells in the cortex (CTX) and the cerebellum (CBL) at week 16. **a** Representative photomicrographs of the cerebellum and cortex immunostained for microglial cells with Iba-1. **b** Quantification of the area occupied by microglial cells in the cortex and the cerebellum of CTL (*white column*) and HFD (*black column*) mice at week 16. Microglial cells were identified using immunohistology in both CNS structures with Iba-1. mRNA expression of **c** CD11b and **d** CD11c, both myeloid lineage markers in the cortex and cerebellum of HFD mice at 16 weeks. **e** Representative photomicrographs of the cortex and cerebellum immunostained for astrocytes with GFAP. **f** Quantification of the area occupied by astrocytes in the cerebellum and the cortex of CTL (*white column*) and HFD (*black column*) mice at week 16. Astrocytes were identified using immunohistology in both CNS structures with GFAP. mRNA expression of **g** GFAP and **h** Serpina3n (an astrogliosis marker) in the cortex and the cerebellum of CTL and HFD mice at week 16. Data are mean ± s.e.m. The standard diet groups were set at 100% for area quantification and at 1 for mRNA expression. The *white columns* represent mice fed a standard diet and the *black columns* represent the mice fed a HFD. One-way ANOVA with Bonferroni’s post test or Kruskal-Wallis test with Dunn’s post test between HFD group and its respective CTL group (*P < 0.05) and between CNS regions (#*P* < 0.05 and ##*P* < 0.01). Scale bar 50 μm

We next sought to characterize further the differences between the cerebellum and the cortex after 16 weeks of HFD feeding by studying the activation state of the microglial cells and astrocytes, two major players in inflammatory processes of the CNS [55, 56]. These cells are known to be involved in the inflammatory processes induced by HFD feeding in the hypothalamus [26, 57]. To this end, we performed an immunohistological analysis of these two cell types in the cerebellum and the cortex of the mice after 16 weeks of HFD feeding. Iba-1 (ionized calcium-binding adapter molecule 1) immunostaining showed no differences in microglial cell activation between the control and the HFD groups, neither in the cerebellum nor in the cortex (Fig. 3a, b). These data are supported by the fact that the mRNA expression of CD11b and CD11c were not affected by the HFD (Fig. 3c, d). On the other hand, immunostaining for astrocytes performed using anti-GFAP (glial fibrillary acidic protein) antibodies showed a larger area occupied by astrocytes in the cerebellum upon HFD feeding, indicating an activated state, an outcome not present in the cortex (Fig. 3e, f). This was further supported by the enhanced GFAP mRNA expression induced by the HFD in the cerebellum compared with CTL mice as well as compared with the cortex of the same animal (Fig. 3g). Finally, to confirm the astrocytes’ state of activation in the cerebellum and the cortex, we studied the expression of an astrogliosis marker Serpina3n (the serine peptidase inhibitor clade A member 3N, also known as alpha-1 antitrypsin) [58, 59]. We found a significant increase of this marker in the cerebellum of HFD mice compared with CTL mice and compared with the cortex of the same mice at week 16 (Fig. 3h). Interestingly, we found no variation of these two astrocyte markers in the cortex between CTL and HFD-fed mice. Our observations seem to point towards the involvement of astrocytes and astrogliosis in the differential inflammatory tone measured in these two CNS regions.

### HFD feeding alters lipid levels in the cerebellum and the cortex at week 16

**Fig. 4:**
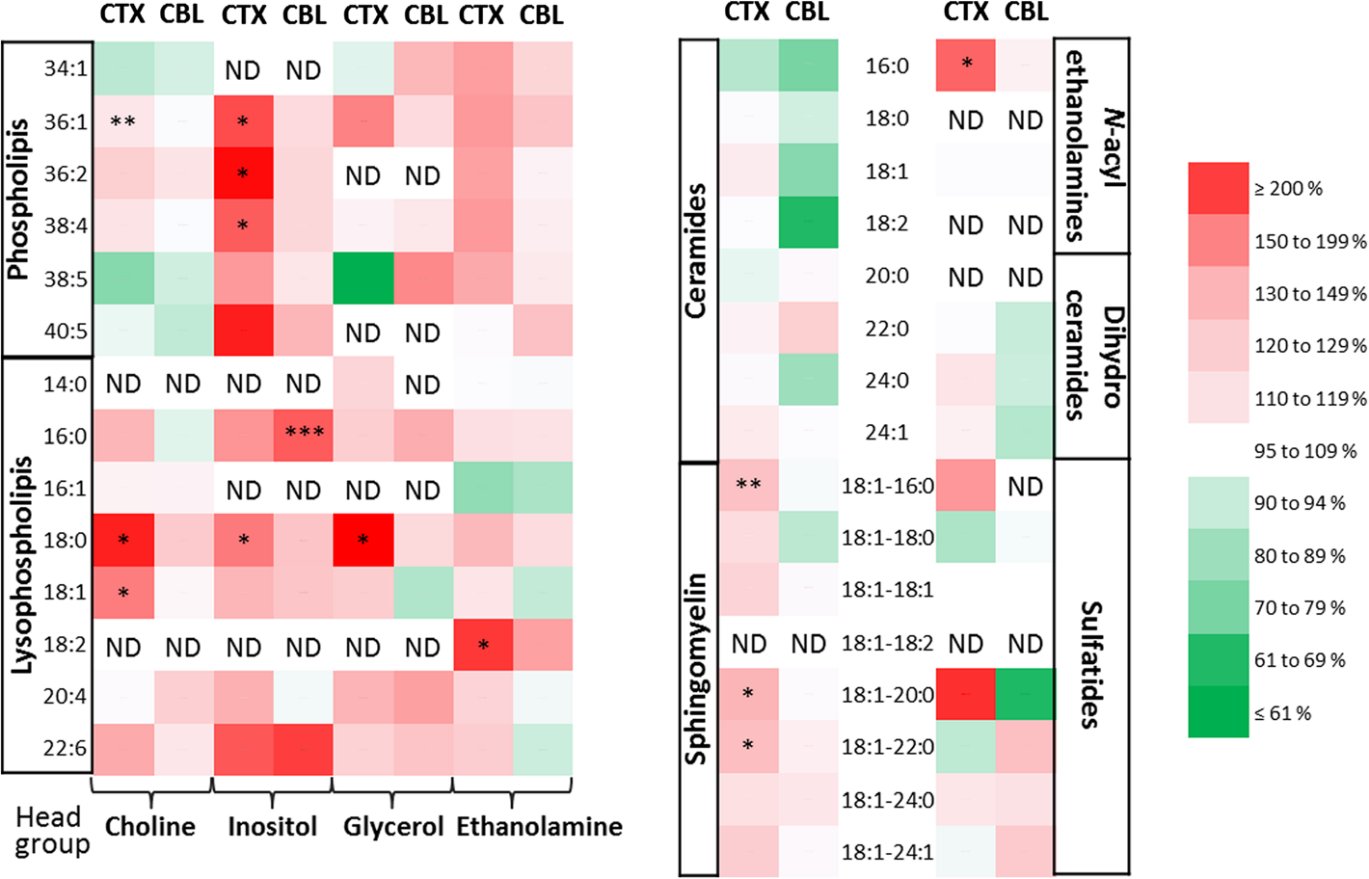
Large-scale lipid screening in the cerebellum (CBL) and the cortex (CTX) at week 16. Phospholipids, lysophospholipids, ceramides, sphingomyelins, *N*-acylethanolamines, dihydroceramides, and sulfatides were measured in the cortex and cerebellum of CTL and HFD mice at week 16 by HPLC-MS. The color *red* represents an increase in lipid levels in the HFD mice compared with the CTL mice. The color *green* represents a decrease in lipid levels in the HFD mice compared with the CTL mice. Numerical data are reported in Additional file 1: Table S2-4. *ND* not detected. For phospholipids, the sum of R1 and R2 acyl chains are indicated, whereas for the other lipids the R acyl chain is mentioned

As mentioned in the introduction, lipids are involved in the control of inflammation. Thus, we decided to perform a broad analysis of the lipids present in the cortex and cerebellum focusing on week 16 because mice displayed a distinct inflammatory tone at this specific time-point. We decided to focus our investigations on ceramides, dihydroceramides, sphingomyelins, sulfatides, and *N*-acylethanolamines as well as phospholipids and lysophospholipids because they are known to be involved in inflammation [60–64]. Globally, the ceramide and dihydroceramide species measured here displayed no variation in the cortex or in the cerebellum. Regarding the sphingomyelin species, their levels were increased in the cortex and showed no variation in the cerebellum. Among the phospholipids and lysophospholipids studied, we found that the levels of phosphatidylinositols, phosphatidylethanolamines, and lysophosphatidylcholines were increased in the cortex upon HFD feeding, while the HFD had much less effect on their levels in the cortex. Of note, we also found that the levels of the anti-inflammatory palmitoylethanolamide were increased in the cortex, but not in the cerebellum, upon HFD feeding (Fig. 4 and Additional file 1: Table S2-4).

### Effects of the identified lipids on primary co-cultured astrocytes and microglia

**Fig. 5:**
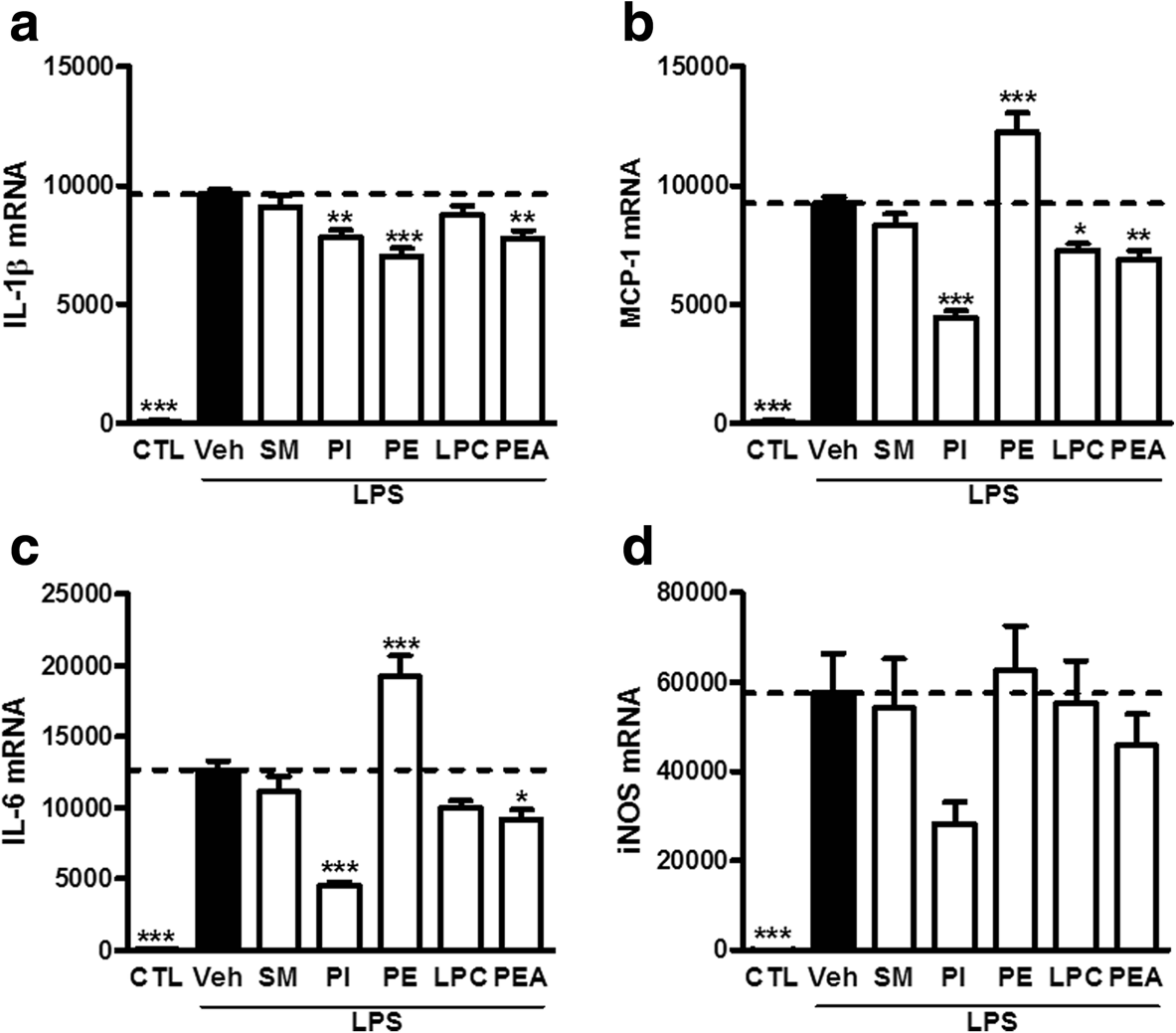
Ex vivo testing of identified lipids. mRNA expression of **a** IL-1β, **b** MCP-1, **c** IL-6, and **d** iNOS in primary co-culture of astrocytes and microglia incubated with sphingomyelins (*SM*), phosphatidylinositols (*PI*), phosphatidylethanolamines (*PE*), lysophosphatidylcholines (*LPC*), or palmitoylethanolamide (*PEA*) prior to LPS activation. Results are expressed relative to the CTL group set at 100 %. The *dotted line* represents the levels obtained for the vehicle-treated LPS-activated group. Results are expressed as mean ± s.e.m. *n* = 2 in triplicate. One-way ANOVA with Dunnett’s post test. *P < 0.05; **P < 0.01; and ***P < 0.001 vs vehicle-treated LPS-activated group

Our HPLC-MS analysis allowed us to identify several lipid classes differently affected by the HFD in the cortex and cerebellum. To determine which of these lipids could potentially be involved in the reduced inflammatory tone found in the cortex, we selected the lipids increased under a HFD in the cortex but decreased (or not affected) in the cerebellum. The lipids that fulfill these criteria are sphingomyelins, phosphatidylinositols, phosphatidylethanolamines, lysophosphatidylcholines, and palmitoylethanolamide. To determine whether these lipids could be in part responsible for the reduced inflammatory tone in the cortex, we tested their effect on microglia–astrocyte-mixed cultures activated by LPS. Incubation of the primary microglia–astrocyte-mixed cultures with LPS induced a strong increase in the expression of inflammatory markers (Fig. 5). While sphingomyelins had no effect on the inflammatory markers, we found that the phosphatidylethanolamines were able to decrease IL-1β expression while they increased IL-6 and MCP-1 expression. Lysophosphatidylcholines were only able to decrease MCP-1 expression while phosphatidylinositols were able to reduce the LPS-induced increase in mRNA expression of IL-1β, IL-6, and MCP-1 (Fig. 5a–c). Interestingly, palmitoylethanolamide was also able to reduce the LPS-induced expression of these three inflammatory markers in these cells. Although palmitoylethanolamide is a known anti-inflammatory and neuroprotective bioactive lipid, this is one of the first reports of the effect of phosphatidylinositols, phosphatidylethanolamines, and lysophosphatidylcholines in such settings. The use of a microglia–astrocyte-mixed culture allowed us to circumvent the bias due to metabolism and blood-brain barrier crossing of the tested lipids. However, additional studies are needed to further support the in vivo role of these lipids in the HFD-induced effects in the cortex.

## Conclusions

In this study, we showed that the peripheral low-grade inflammation induced by a HFD does not affect the different regions of the CNS in the same way and that this inflammatory tone is also time-dependent. In this particular setting, we were able to identify the potential involvement of glial cells and, more precisely, astrocytes. Interestingly, these CNS inflammatory cells are also involved in the control of the blood-brain barrier permeability. In the cerebellum, we found activated astrocytes and increased expression of claudin 1 and occludin. This increased expression of tight junction proteins could be a potential mechanism aiming at restoring the blood-brain barrier integrity in order to reduce the inflammatory insult evidenced in this CNS area. These changes were absent in the cortex where the inflammatory tone was similar to the one of chow-fed mice. These findings further support that the low-grade inflammatory tone resulting from a HFD differentially affects the two specific regions of the CNS studied.

We further characterized the specific micro-environment in these two CNS areas by measuring levels of bioactive lipids. Upon testing on primary co-culture of microglia and astrocytes, we identified phosphatidylinositols, lysophosphatidylcholines, and PEA as potential anti-inflammatory compounds as they were increased in the cortex of HFD mice at week 16 and were also able to decrease the expression of pro-inflammatory markers ex vivo. This study demonstrates that not all CNS regions are equal when facing obesity-driven inflammatory insults. Finally, this work paves the way for further research revolving around the effects of the identified lipids as anti-inflammatory compounds in obesity and other inflammatory settings influencing the CNS.

## Additional files

Additional file 1: Table S1. Composition of the diets used in the diet-induced obesity model. **Table S2.** High-fat diet-induced changes in phospholipid and lysophospholipid cortical levels. **Table S3**. High-fat diet-induced changes in phospholipid and lysophospholipid cerebellar levels. **Table S4.** High-fat diet-induced changes in lipid levels. (PDF 354 kb) Additional file 2: Figure SI 1. Weight of the mice on standard (empty dots) and high-fat (full dots) diets in the different groups after (a) 1 week, (b) 2 weeks, (c) 4 weeks, (d) 6 weeks, (e) 8 weeks, and (f) 16 weeks. Data are mean ± s.e.m.; two-way ANOVA with post hoc Bonferroni test between HFD group and its CTL group **P* < 0.05; ***P* < 0.01; and ****P* < 0.001. **Figure SI 2.** Weight of the (a) subcutaneous adipose tissue, (b) epididymal adipose tissue, and (c) visceral adipose tissue at the selected time-points. Data are mean ± s.e.m.; Student’s *t* test or Mann-Whitney test between each HFD group (Black columns) and its respective CTL group (white columns) **P* < 0.05; ***P* < 0.01; and ****P* < 0.001. **Figure SI 3.** Total plasma cholesterol measured at (a) week 1 and (b) week 16. (c) Inflammatory markers measured in the plasma at week 16. Data are mean ± s.e.m.; Student’s *t* test between the HFD group and its CTL group **P* < 0.05; ****P* < 0.001. **Figure SI 4.** mRNA relative expression of inflammatory markers in the brainstem at (a) 1 week and (b) 16 weeks. The expression level in the control group is set at 100. Data are mean ± s.e.m.; Student’s *t* test between the HFD group and its CTL group **P* < 0.05; ***P* < 0.01; and ****P* < 0.001. **Figure SI 5.** mRNA relative expression of junction proteins of the blood-brain barrier at week 16 in the cortex and the cerebellum. The expression levels in the control groups were set at 100. Data are mean ± s.e.m. The white columns represent mice fed a standard diet and the black columns represent the mice fed a HFD. One-way ANOVA with Bonferroni’s post test and between HFD group and its respective CTL group **P* < 0.05 and ****P* < 0.001 and between CNS regions #*P* < 0.05 and ###*P* < 0.001. (PDF 67 kb)

## Abbreviations

CBL: Cerebellum
COX-2: Cyclooxygenase-2
CTX: Cortex
EAT: Epididymal adipose tissue
GFAP: Glial fibrillary acidic protein
HFD: High-fat diet
Iba-1: Ionized calcium-binding adapter molecule 1
IL-1β: Interleukin-1β
iNOS: Inducible nitric oxide synthase
LBP: LPS-binding protein
LPC: Lysophosphatidylcholine
LPS: Lipopolysaccharides
MCP-1: Monocyte chemoattractant protein 1 (or CCL2)
PE: Phosphatidylethanolamine
PEA: Palmitoylethanolamide
PI: Phosphatidylinositol
SAT: Subcutaneous adipose tissue
Serpina3n: Serine peptidase inhibitor, clade A, member 3N (or alpha 1-antitrypsin)
SM: Sphingomyelin
TNFα: Tumor necrosis factor α
VAT: Visceral adipose tissue
ZO-1: Zonula occludens-1
