# Supplementary material for: High-fat diet feeding differentially affects the development of inflammation in the central nervous system
Source: J Neuroinflammation. 2016 Aug 26;13(1):206. doi: 10.1186/s12974-016-0666-8 (PMC5002131; doi:10.1186/s12974-016-0666-8)
Supplement: Additional file 1: Table S1. — Composition of the diets used in the diet-induced obesity model. Table S2. High-fat diet-induced changes in phospholipid and lysophospholipid cortical levels. Table S3. High-fat diet-induced changes in phospholipid and lysophospholipid cerebellar levels. Table S4. High-fat diet-induced changes in lipid levels. (PDF 354 kb) [file 12974_2016_666_MOESM1_ESM.pdf]

Table S1. Composition of the diets used in the diet-induced obesity model

Nutrient percentage (of weight (g)) provided by fat, carbohydrates and proteins as well as fatty acid composition of the diets according to the manufacturer.

% gm	CTL diet (AIN-93M)	High fat diet (D12492)
Fat	4	35
Carbohydrates	73.1	26
Proteins	14.2	26
**Fatty acid profile**		
Saturated (g)	5.7	81.5
Monounsaturated (g)	9.7	91.5
Polyunsaturated (g)	24.5	81.5

Table S2. **High-fat diet-induced changes in phospholipid and lysophospholipid cortical levels**

		acyl chains	head group			
			Choline	Inositol	Glycerol	Ethanolamine
CORTEX	Phospholipids	34:1	90 ± 6	N.D.	96 ± 21	150 ± 20
		36:1	112 ± 3 **	194 ± 15 *	165 ± 47	153 ± 13
		36:2	124 ± 11	229 ± 47*	N.D.	149 ± 24
		38:4	114 ± 13	185 ± 13 *	105 ± 18	153 ± 25
		38:5	82 ± 9	153 ± 11	61 ± 21	144 ± 25
		40:5	97 ± 12	219 ± 39	N.D.	101 ± 25
	Lysophospholipids	14:0	N.D.	N.D.	121 ± 13	100 ± 11
		16:0	137 ± 22	156 ± 11	151 ± 6	116 ± 13
		16:1	105 ± 28	N.D.	N.D.	84 ± 7
		18:0	217 ± 42 *	169 ± 19 *	233 ± 28 *	136 ± 28
		18:1	167 ± 27 *	137 ± 9	125 ± 6	113 ± 15
		18:2	N.D.	N.D.	N.D.	205 ± 21 *
		20:4	101 ± 20	141 ± 11	139 ± 19	121 ± 13
		22:6	144 ± 24	188 ± 67	123 ± 9	125 ± 15

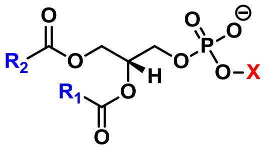

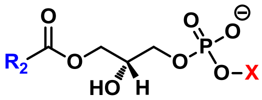

HPLC-MS relative quantification of phospholipids and lysophospholipids in the cortex of high-fat diet fed-mice and control diet-fed mice at week 16. The levels in control mice were set at 100%. Data are mean ± s.e.m. One way ANOVA with Bonferroni's post-test or Kruskal-Wallis test with Dunns post-test *P<0.05 and **P<0.01.

Table S3. High-fat diet-induced changes in phospholipid and lysophospholipid cerebellar levels

		acyl chain(s)	Head group			
			Choline	Inositol	Glycerol	Ethanolamine
CEREBELLUM	Phospholipids	34:1	94 ± 1	137 ± 30	137 ± 30	121 ± 17
		36:1	100 ± 2	118 ± 27	118 ± 26	130 ± 11
		36:2	113 ± 3	120 ± 20	N.D.	106 ± 14
		38:4	100 ± 3	120 ± 21	111 ± 24	108 ± 12
		38:5	93 ± 4	112 ± 14	160 ± 45	111 ± 18
		40:5	90 ± 2	137 ± 27	N.D.	131 ± 16
	Lysophospholipids	14:0	N.D.	N.D.	N.D.	99 ± 23
		16:0	96 ± 13	186 ± 18 ***	142 ± 32	114 ± 15
		16:1	106 ± 14	N.D.	N.D.	87 ± 17
		18:0	127 ± 30	130 ± 14	119 ± 22	117 ± 17
		18:1	104 ± 15	129 ± 8	88 ± 3	91 ± 13
		18:2	N.D.	N.D.	N.D.	149 ± 25
		20:4	124 ± 15	99 ± 10	149 ± 25	98 ± 14
		22:6	112 ± 19	201 ± 26	130 ± 20	93 ± 13

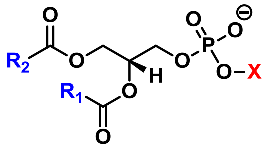

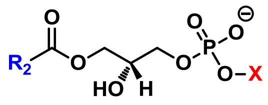

HPLC-MS relative quantification of phospholipids and lysophospholipids in the cerebellum of high-fat diet fed-mice and control diet-fed mice at week 16. The CTL levels were set at 100%. Data are mean ± s.e.m. One way ANOVA with Bonferroni's post-test or Kruskal-Wallis test with Dunns post-test ***P<0.001

Table S4. High-fat diet-induced changes in lipid levels

		CTX	CBL
***N*-acylethanolamines**			
	16:0	180 ± 48 *	107 ± 5
	18:1	100 ± 13	101 ± 13
	20:4	128 ± 16	123 ± 10
**CERAMIDES**			
	16:0	89 ± 10	80 ± 17
	18:0	100 ± 10	93 ± 4
	18:1	109 ± 23	82 ± 5
	18:2	101 ± 6	66 ± 11
	20:0	97 ± 11	102 ± 7
	22:0	106 ± 17	124 ± 8
	24:0	102 ± 19	85 ± 5
	24:1	110 ± 14	100 ± 6
**DIHYDROCERAMIDES**			
	20:0	101 ± 21	92 ± 8
	24:0	113 ± 23	92 ± 13
	24:1	107 ± 21	89 ± 10
**Sulfatides**	18:1–16:0	154 ± 32	N.D.
	18:1–18:0	88 ± 15	99 ± 15
	18:1–18:1	N.D.	N.D.
	18:1–18:2	N.D.	N.D.
	18:1–20:0	209 ± 73	66 ± 18
	18:1–22:0	90 ± 25	132 ± 11
	18:1–24:0	112 ± 21	115 ± 12
	18:1–24:1	98 ± 10	127 ± 12
**Sphingomyelins**	18:1–16:0	131 ± 10 **	99 ± 5
	18:1–18:0	117 ± 15	90 ± 2
	18:1–18:1	122 ± 5	102 ± 8
	18:1–18:2	N.D.	N.D.
	18:1–20:0	138 ± 17 *	101 ± 3
	18:1–22:0	133 ± 14 *	108 ± 3
	18:1–24:0	115 ± 11	112 ± 2
	18:1–24:1	125 ± 10	102 ± 4

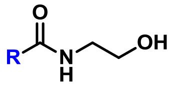

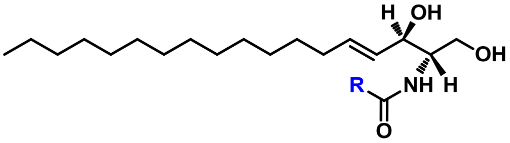

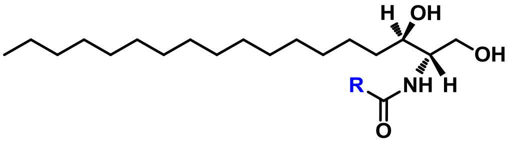

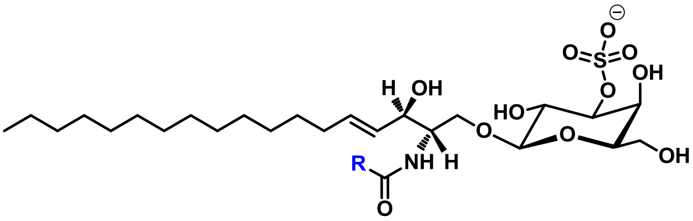

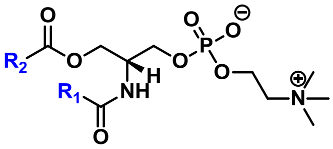

HPLC-MS relative quantification ceramides, dihydroceramides, sulfatides and sphingomyelins in the cortex and the cerebellum of high-fat diet fed-mice and control diet-fed mice at week 16. The CTL levels were set at 100%. Data are mean ± s.e.m. One way ANOVA with Bonferroni's post-test or Kruskal-Wallis test with Dunns post-test *P<0.05 and **P<0.01
