# Supplementary material for: High-fat diet feeding differentially affects the development of inflammation in the central nervous system
Source: J Neuroinflammation. 2016 Aug 26;13(1):206. doi: 10.1186/s12974-016-0666-8 (PMC5002131; doi:10.1186/s12974-016-0666-8)
Supplement: Additional file 2: Figure SI 1. — Weight of the mice on standard (empty dots) and high-fat (full dots) diets in the different groups after (a) 1 week, (b) 2 weeks, (c) 4 weeks, (d) 6 weeks, (e) 8 weeks, and (f) 16 weeks. Data are mean ± s.e.m.; two-way ANOVA with post hoc Bonferroni test between HFD group and its CTL group *P < 0.05; **P < 0.01; and ***P < 0.001. Figure SI 2. Weight of the (a) subcutaneous adipose tissue, (b) epididymal adipose tissue, and (c) visceral adipose tissue at the selected time-points. Data are mean ± s.e.m.; Student’s t test or Mann-Whitney test between each HFD group (Black columns) and its respective CTL group (white columns) *P < 0.05; **P < 0.01; and ***P < 0.001. Figure SI 3. Total plasma cholesterol measured at (a) week 1 and (b) week 16. (c) Inflammatory markers measured in the plasma at week 16. Data are mean ± s.e.m.; Student’s t test between the HFD group and its CTL group *P < 0.05; ***P < 0.001. Figure SI 4. mRNA relative expression of inflammatory markers in the brainstem at (a) 1 week and (b) 16 weeks. The expression level in the control group is set at 100. Data are mean ± s.e.m.; Student’s t test between the HFD group and its CTL group *P < 0.05; **P < 0.01; and ***P < 0.001. Figure SI 5. mRNA relative expression of junction proteins of the blood-brain barrier at week 16 in the cortex and the cerebellum. The expression levels in the control groups were set at 100. Data are mean ± s.e.m. The white columns represent mice fed a standard diet and the black columns represent the mice fed a HFD. One-way ANOVA with Bonferroni’s post test and between HFD group and its respective CTL group *P < 0.05 and ***P < 0.001 and between CNS regions #P < 0.05 and ###P < 0.001. (PDF 67 kb) [file 12974_2016_666_MOESM2_ESM.pdf]

Supplemental Figure 1

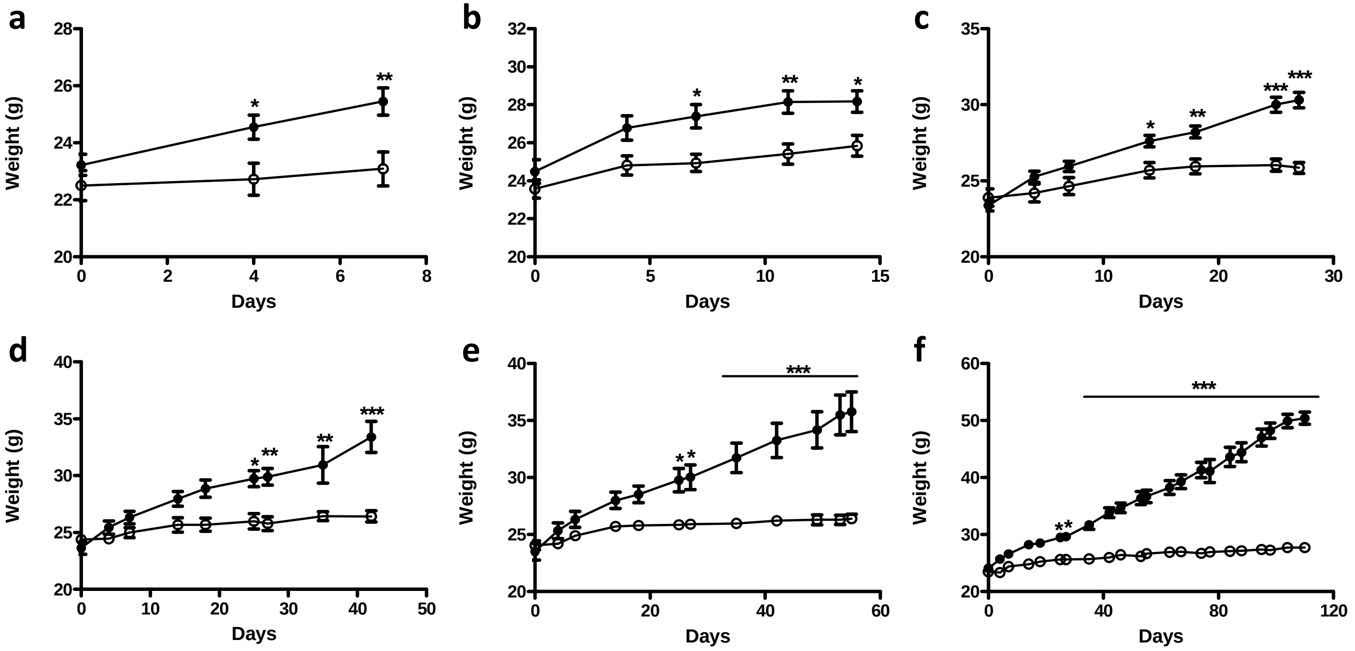

SI 1. Weight of the mice on standard (empty dots) and high-fat (full dots) diets in the different groups after (**a**) 1 week, (**b**) 2 weeks, (**c**) 4 weeks, (**d**) 6 weeks, (**e**) 8 weeks and (**f**) 16 weeks. Data are mean ± s.e.m.; two-way ANOVA with post hoc Bonferroni test between HFD group and its CTL group *P<0.05; **P<0.01 and ***P<0.001

Supplemental Figure 2

Guillemot-Legris et al.

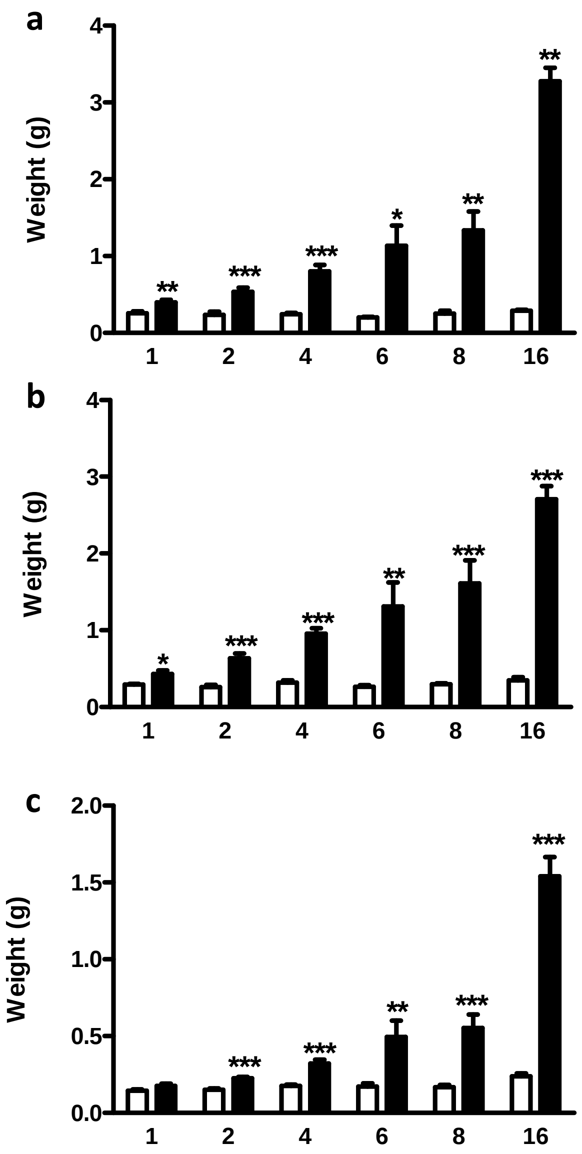

SI 2. Weight of the (**a**) subcutaneous adipose tissue, (**b**) epididymal adipose tissue and (**c**) visceral adipose tissue at the selected time-points. Data are mean ± s.e.m.; Student's t-test or Mann-Whitney test between each HFD group (~~full bars~~) and its respective CTL group (~~empty bars~~) *P<0.05; **P<0.01 and ***P<0.001.

Supplemental Figure 3

Guillemot-Legris et al.

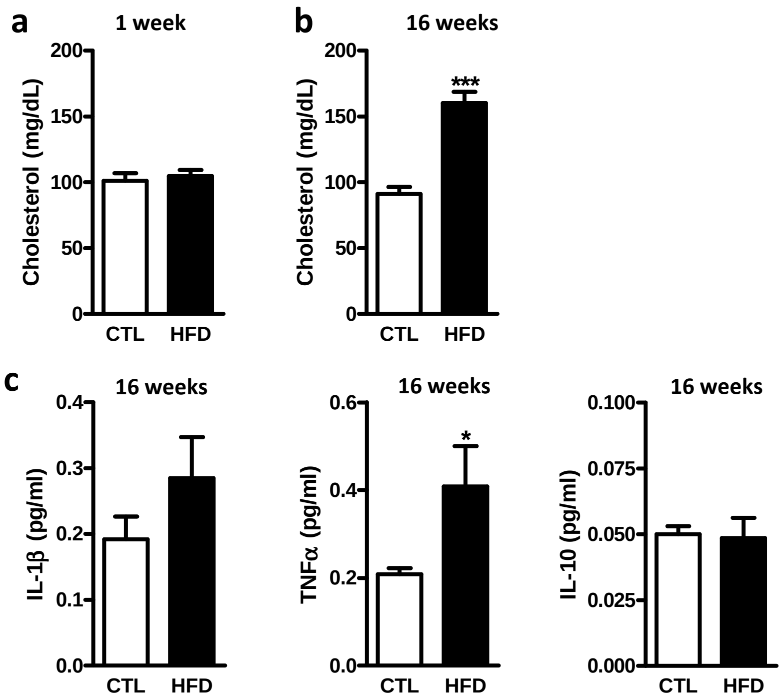

SI 3. Total plasma cholesterol measured at (**a**) ~~week 1and~~ (**b**) week 16. (**c**) Inflammatory markers measured in the plasma at week 16. Data are mean ± s.e.m.; Student's t-test between the HFD group and its CTL group *P<0.05 ***p<0.001.

Supplemental Figure 4

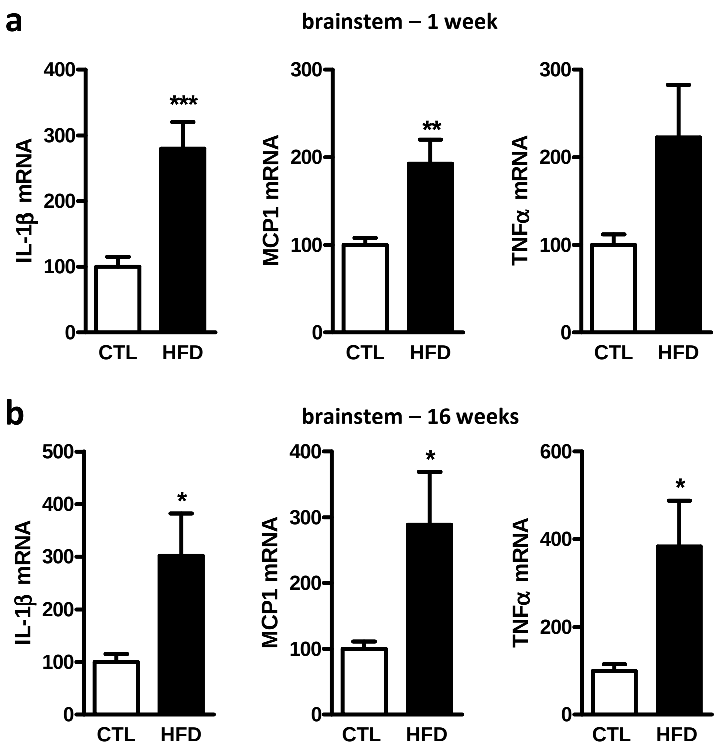

SI 4. mRNA relative expression of inflammatory markers in the brainstem at (**a**) 1 week and (**b**) 16 weeks. The expression level in the control group is set at 100. Data are mean ± s.e.m.; Student's t-test between the HFD group and its CTL group *P<0.05; **P<0.01 and ***P<0.001.

Supplemental Figure 5

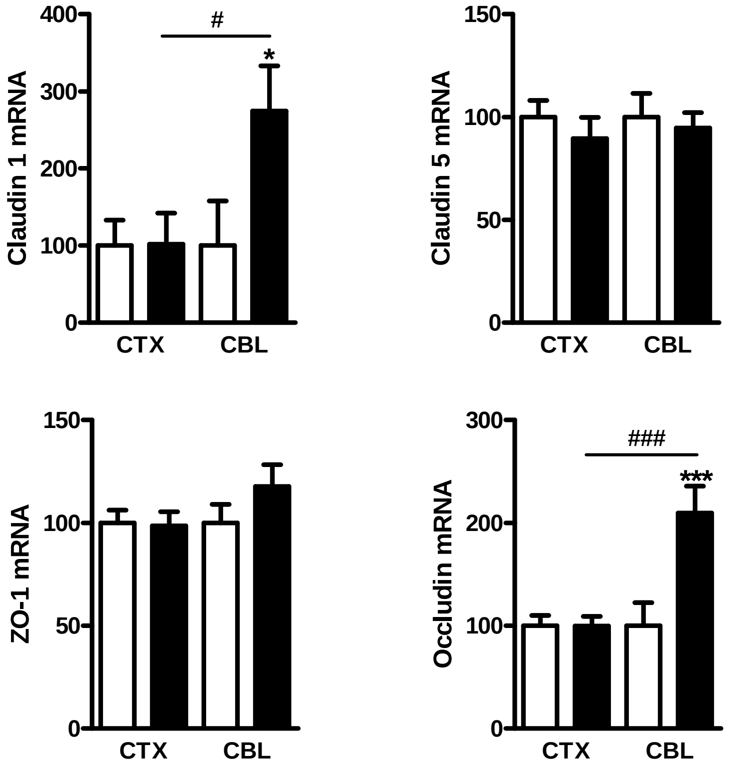

SI 5. mRNA relative expression of junction proteins of the blood brain barrier at week 16 in the cortex and the cerebellum. The expression levels in the control ~~group~~ were set at 100. Data are mean ± s.e.m. The white columns represent mice fed a standard diet and the black columns represent the mice fed a HFD. One way ANOVA with Bonferroni's post-test and between HFD group and its respective CTL group *P<0.05 and ***P<0.001 and between CNS regions #P<0.05 and ###P<0.001
